# Porcine circovirus 2 (PCV-2) genetic variability under natural infection scenario reveals a complex network of viral quasispecies

**DOI:** 10.1038/s41598-018-33849-2

**Published:** 2018-10-19

**Authors:** Florencia Correa-Fiz, Giovanni Franzo, Anna Llorens, Joaquim Segalés, Tuija Kekarainen

**Affiliations:** 1 Centre de Recerca en Sanitat Animal (CReSA, IRTA-UAB), IRTA, Bellaterra, Spain; 2 Department of Animal Medicine, Production and Health (MAPS), University of Padua, Legnaro, PD Italy; 3 Departament de Sanitat i Anatomia Animals, Facultat de Veterinària, UAB, Bellaterra, Spain; 4 Present Address: Kuopio Center for Gene and Cell Therapy, Microkatu 1, Kuopio, Finland

## Abstract

Porcine circovirus 2 (PCV-2) is a virus characterized by a high evolutionary rate, promoting the potential emergence of different genotypes and strains. Despite the likely relevance in the emergence of new PCV-2 variants, the subtle evolutionary patterns of PCV-2 at the individual-host level or over short transmission chains are still largely unknown. This study aimed to analyze the within-host genetic variability of PCV-2 subpopulations to unravel the forces driving PCV-2 evolution. A longitudinal weekly sampling was conducted on individual animals located in three farms after the first PCV-2 detection. The analysis of polymorphisms evaluated throughout the full PCV-2 genome demonstrated the presence of several single nucleotide polymorphisms (SNPs) especially in the genome region encoding for the capsid gene. The global haplotype reconstruction allowed inferring the virus transmission network over time, suggesting a relevant within-farm circulation. Evidences of co-infection and recombination involving multiple PCV-2 genotypes were found after mixing with pigs originating from other sources. The present study demonstrates the remarkable within-host genetic variability of PCV-2 quasispecies, suggesting the role of the natural selection induced by the host immune response in driving PCV-2 evolution. Moreover, the effect of pig management in multiple genotype coinfections occurrence and recombination likelihood was demonstrated.

## Introduction

Porcine circovirus 2 (PCV-2) is a non-enveloped single-stranded DNA virus with a small circular genome of 1766–1768 bp^1^. The PCV-2 genome consists in at least four major open reading frames (ORFs) encoding the replicase protein (ORF1), the viral capsid protein (ORF2) and proteins with either apoptotic (ORF3) or anti-apoptotic capacities (ORF4)^2,3^. PCV-2 belongs to the genus Circovirus from the Circoviridae family, together with two other porcine circoviruses, PCV-1^4^ and PCV-3^5^. Although its small size, PCV-2 is of great importance in the global swine industry economy, since it is the primary causative agent of porcine circovirus diseases (PCVD)^6^, especially PCV-2 systemic disease (PCV-2-SD, formerly known as postweaning multisystemic wasting syndrome), which causes severe losses in intensive pig production worldwide^7^. PCV-2 has a complex epidemiology, featured by an effective among-animal transmission due to the different shedding routes and the long viral persistence in infected animals^8^.

Four main genotypes have been determined based on the sequence analysis of the whole genome and cap gene, with PCV-2a, PCV-2b, PCV-2c and PCV-2d being globally distributed^9^. More recently, two additional genotypes have been proposed^10,11^. ORF2 is particularly relevant because of not only a higher genetic heterogeneity, facilitating viral classification and molecular epidemiology studies, but also for its relevance in the host immune response^12–14^. The genetic variability of this virus has been demonstrated to be remarkable, with the highest evolutionary rate among DNA viruses^15^. Several mutations, particularly in the ORF2 region have been reported worldwide with different outcomes both *in vivo* and *in vitro*, with recombinant strains being also relevant in the distribution of different genotypes^12^. Moreover, during the last decades, novel variants of PCV-2 have been identified^16,17^, increasing the interest in monitoring the possibility of emergence of new strains and the role of these circulating strains in PCV-2 evolution. The evolutionary pathways of this virus have shown to be particularly relevant regarding the clinical manifestations occurring through time^12^. Despite the likely relevance in the emergence of new PCV-2 variants, the subtle evolutionary patterns of PCV-2 at individual-host level or over short transmission chains are still largely unknown. The aim of this study was to analyze the genetic variability of PCV-2 subpopulations at individual pig level in different farms and unravel the forces driving PCV-2 evolution *in vivo*.

## Results

A total of 20 piglets from each of three different Spanish farms analyzed (farms A-C) were sampled and checked weekly to detect their first evidence of PCV-2 infection in life. Once detected, 5 PCV-2-positive animals per farm were sampled weekly for 4 weeks post infection (wpi) and the full PCV-2 genome was sequenced using next generation technology. A summary of PCV-2 positive animal sampling and sequencing results is reported in Supplementary Fig. S1. Briefly, a mean coverage greater than approximately 300 reads was obtained for all the pigs included in the study, with the only exception of pig 737 at week 8 (farm C) and 776 at week 11 (farm A).

**Figure 1:**
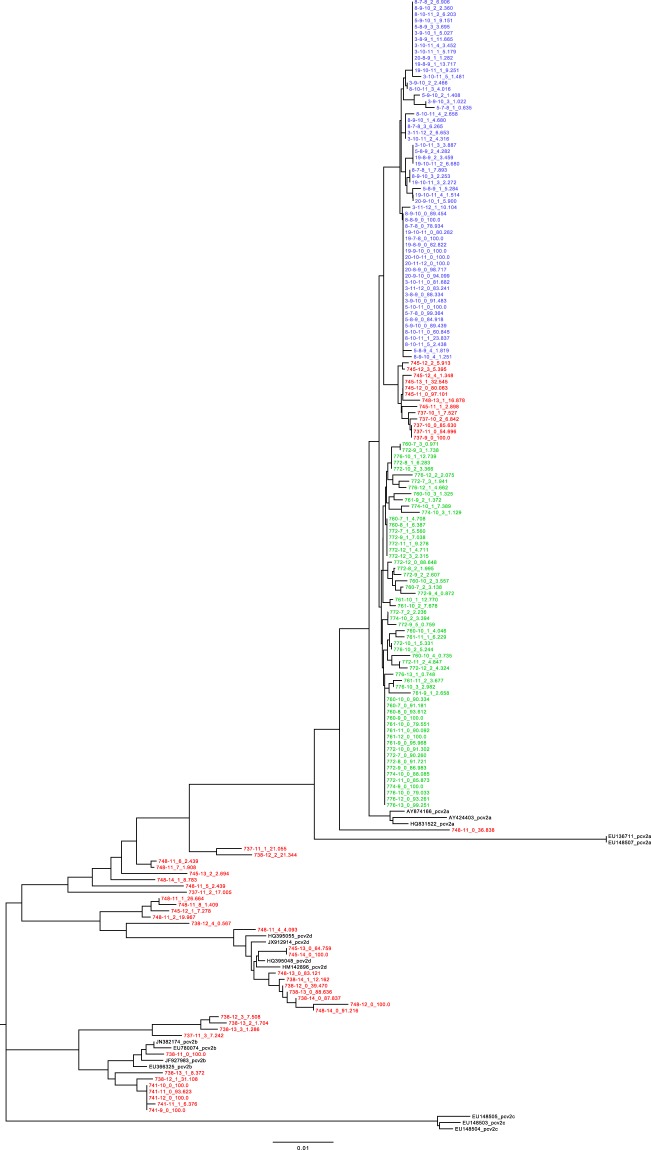
Maximum likelihood phylogenetic tree reconstructed using the ORF2 gene of estimated haplotypes and the reference sequences proposed by Franzo *et al*., 2015. Individual haplotypes have been named using the following scheme: AnimalID-SamplingWeek_HaplotypeNumber_HaplotypePrevalence. Farm A, B and C have been color-coded in green, blue and red, respectively.

The study of viral global haplotypes performed at individual animal level (Fig. 1) revealed the presence of remarkably different scenarios among the farms. Particularly, all strains sampled from both farm A and B formed two distinct farm-specific clades belonging to the PCV-2a genotype. In farm C, PCV-2b genotype was the only one detected until 11 weeks of age. At that age, all pigs were relocated within fattening facilities remaining exclusively in contact with pigs from the same origin except for farm C where they were mixed with animals from different herds. After this event, the scenario became much more complex in farm C and strains belonging to different genotypes (PCV-2a, PCV-2b and PCV-2d) were identified in different animals at different time points. Additionally, several co-infections with multiple genotypes were observed and both tree topology and recombination analysis suggested the presence of several recombination events. Based on these results, the experiment was further split into two studies. Farms A and B were selected to evaluate the pattern of PCV-2 genetic variability within and between animals over time (Study 1). The presence of multiple genotypes impeded this analysis in farm C since most of the variability would be attributable to the long term evolution separating the PCV-2 genotypes. At the same time, this genetic distance facilitates the differentiation of different strains despite the unavoidable limits inherent to NGS data analysis. Consequently, farm C was selected to investigate the within-farm spreading pattern of PCV-2 and its consequences (Study 2).

### Study 1: Analysis of PCV-2 genetic variability over time

**Figure 2:**
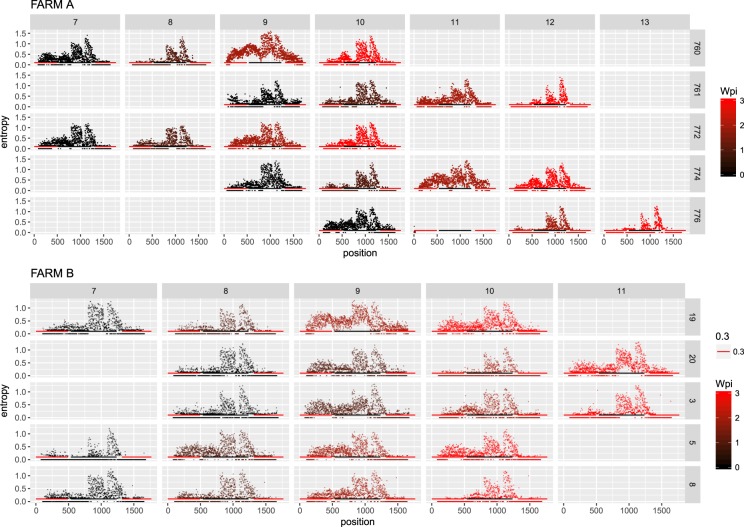
Entropy value at each genome position are reported for farm A (top) and farm B (bottom). Data for different animal and week of age have been reported separately. Week post infection has been color coded. The ORF1 and ORF2 have been respectively represented as red and black line in the lower part of each graph.

**Figure 3:**
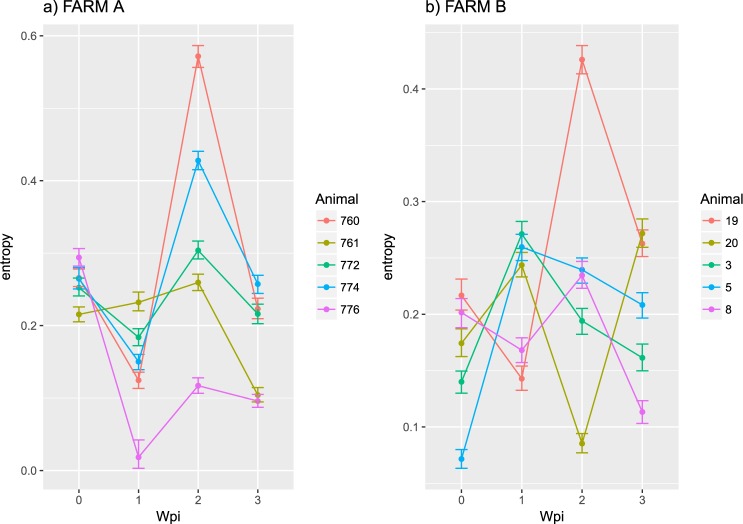
Mean entropy (point) and 95 confidence intervals (error-bars; calculated using bootstrap) are reported for each animal at different wpi.

**Figure 4:**
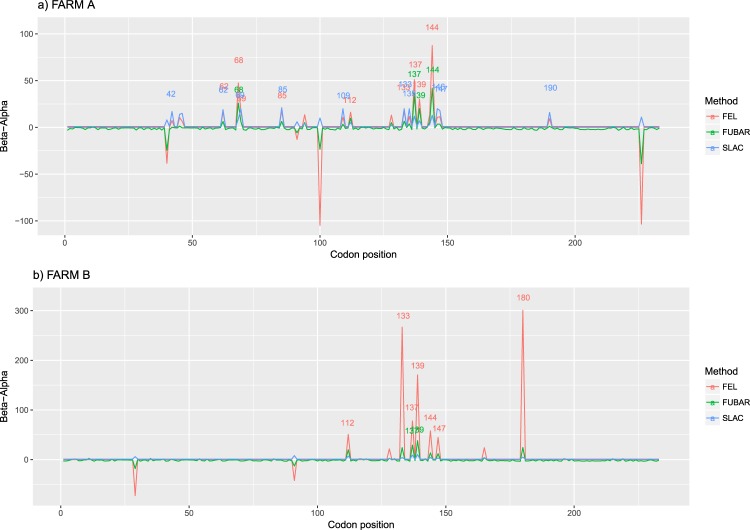
Estimate of selective pressures acting on the *Cap* gene. Normalized dN–dS is displayed for each codon position. Results of SLAC, FUBAR and FEL method have been color-coded.

**Figure 5:**
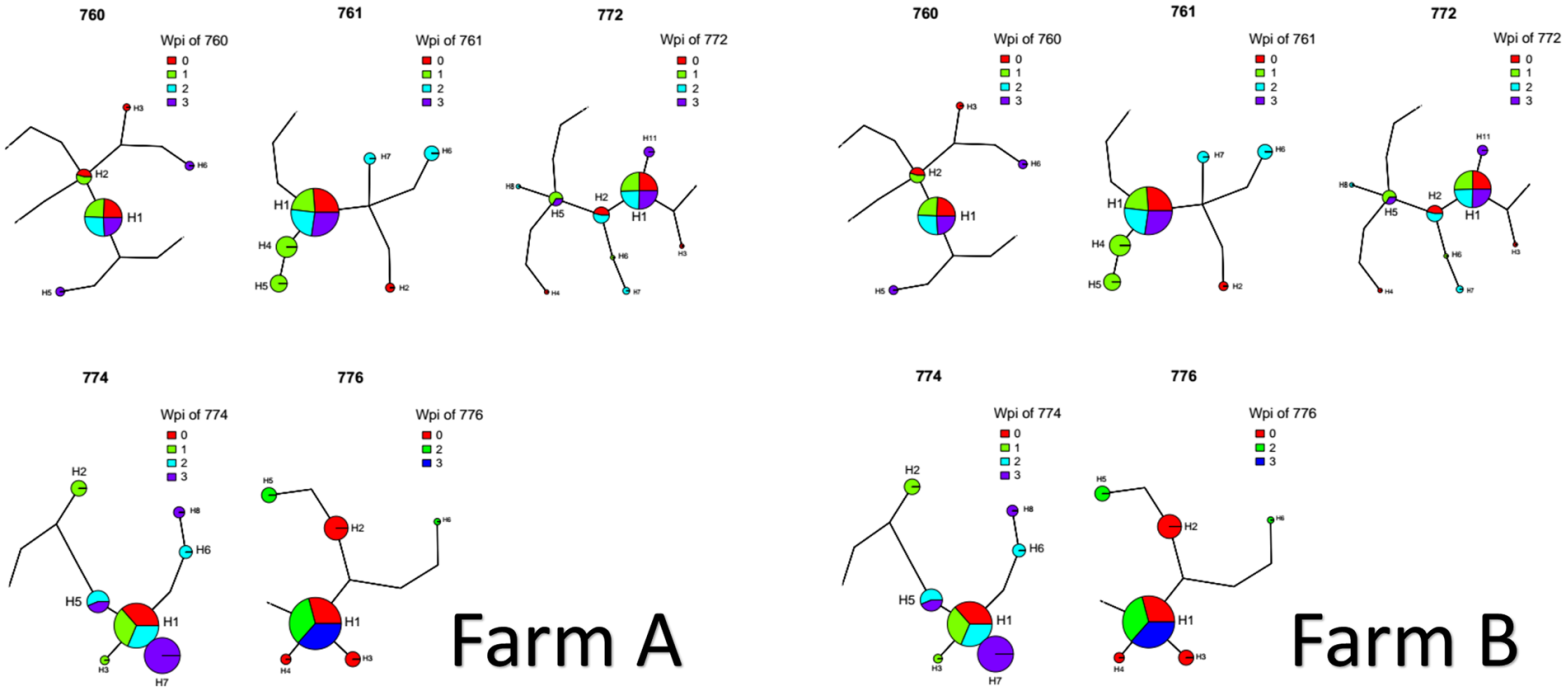
Haplotype network drawn for each animal (animal ID is reported above the graph). The size of the circle is proportional to the prevalence of the haplotype while the different week post infection have been color coded. The five animals from farm A are represented on the left while the ones from farm B are represented on the right.

Entropy was calculated throughout the whole genome to obtain a statistical summary representing the genetic variability at each genome position. The overall pattern analysis evidenced higher variability in the ORF2 region compared to the rest of the genome, irrespective of the farm, animal and sampling date, as depicted in Fig. 2. A comparable scenario was observed when nucleotide frequencies were considered. Despite the more stringent single nucleotide variation (SNV) acceptance criteria, several subpopulations were detected both in farm A and B (Supplementary Fig. S2). Although some SNV were identified in the ORF1 region, most of them affected the ORF2. The average entropy displayed different trends when analyzed in the two farms under study. In farm A, there was a fluctuating pattern in overall variability, which showed a decreased tendency at 1 wpi, increased at the following sampling week and, finally, decreased by the end of the study (Fig. 3a). However, the scenario was much more complex in farm B, where entropy patterns over time differed remarkably among animals and a consistent trend could hardly be identified (Fig. 3b). In both farms, individual animals showed peculiar patterns in viral genetic heterogeneity, with some pigs evidencing major fluctuations in entropy values over time while an essentially constant variability was detected in others through the whole study period. Despite these differences, some similarities were observed in the analysis of selective pressures acting on the Cap gene of those strains. Particularly, an area under diversifying selection was observed approximately between AA 130 and 150 in sequences from both farms, while some evidences of a certain selective diversifying selection were observed to act on other protein regions only for sequences obtained from farm A (Fig. 4). Nevertheless, it must be stressed that only for some specific positions the deviation from neutral selection was statistically significant. Particularly, the MEME method, able to detect episodic diversifying selection, detected evidences of positive selection in AA position 137 (p-value 0.08) in farm A and at codons 133 (p-value 0.10) and 180 (p-value 0.08) in farm B. A summary of codons detected under diversifying selection using other methods is reported in Table 1 and depicted in the protein 3D-structure in Supplementary Fig. S3 (see also Supplementary Video). Similarly, the haplotype network analysis performed in the ORF2 gene demonstrated a fairly repeatable dynamics among different farms and animals, characterized by the presence of a major haplotype persisting for the whole period of time (except for animal 774 at 3 wpi) and minor ones emerging over time. Some of those haplotypes were detected only once, while others were able to persist for longer periods of time, as shown in Fig. 5. No evidences of a relevant correlation between humoral immune response and PCV-2 variability were apparent either globally or in sub-genomic regions (Supplementary Figs S4 and S5).

**Table 1:** Sites under diversifying selection in farm A (upper) and farm B (lower).

POSITION	FEL		FUBAR		SLAC	
	beta-alpha	p-value	beta-alpha	Pos. Prob.	beta-alpha	p-value
68	47.485	0.187	26.094	0.955	26.007	0.297
112	16.192	0.275	9.704	0.904	15.625	0.444
137	50.997	0.057	34.919	0.988	46.875	0.087
139	30.033	0.238	20.113	0.951	19.558	0.509
144	87.469	0.111	41.912	0.977	54.687	0.303
112	50.779	0.261	19.922	0.935	41.509	0.444
133	266.901	0.077	24.066	0.904	20.754	0.666
137	77.874	0.169	29.263	0.966	62.264	0.087
139	170.577	0.214	38.084	0.962	63.036	0.509
180	300.844	0.055	24.432	0.911	37.907	1

### Study 2. Spreading pattern of PCV-2 and its consequences

**Figure 6:**
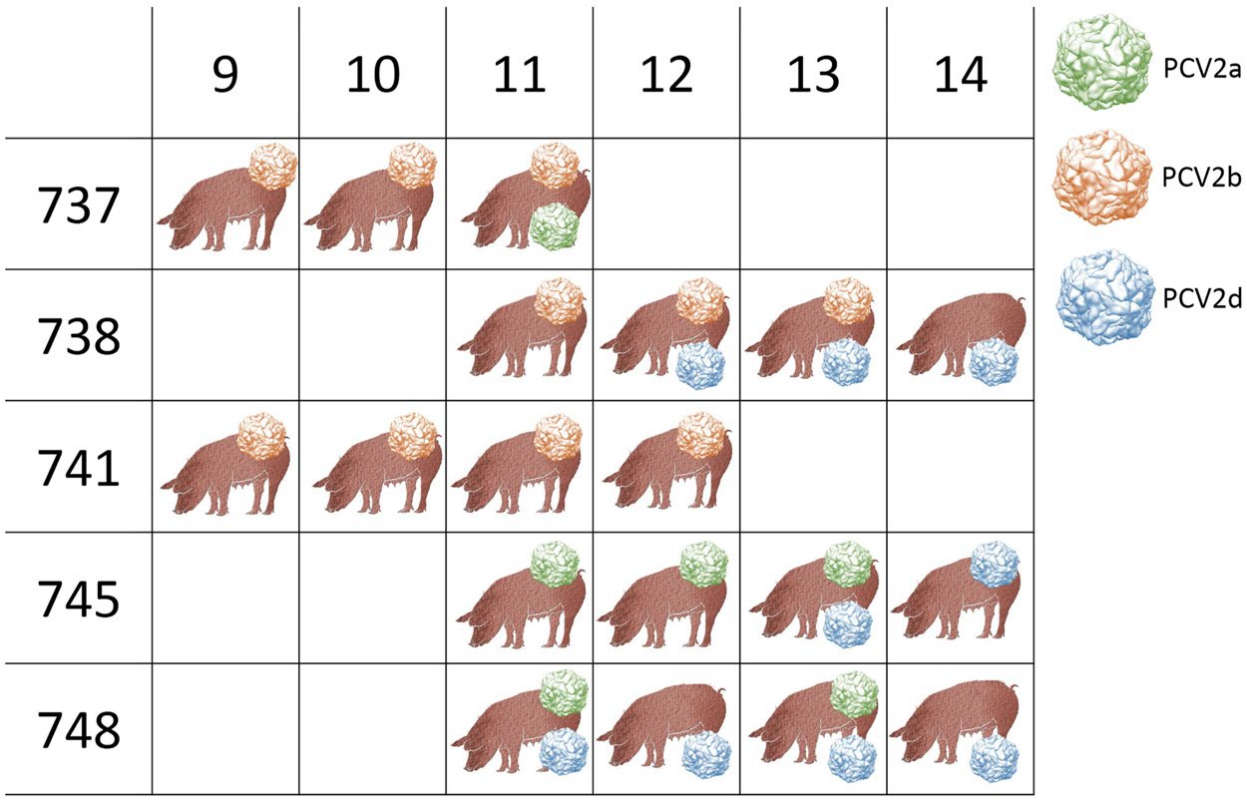
Summary of genotypes (color-coded) detected in different animals at different weeks of age. All considered animals originated from farm C.

The analysis of viral circulation within the herd showed a remarkable variation in the farm C epidemiological scenario after the mixing with animals from different origins. While two animals were already infected with PCV-2b in the origin farm, the other became positive with PCV-2a or PCV-2d after the mixing (Fig. 6). In 4 out of 5 animals, co-infections were detected, persisting for more than two weeks in 3 out of 4 pigs (Fig. 6). The co-infection presence was further confirmed by the SNV analysis using LoFreq, which showed an extremely high SNV frequency in some animals (Supplementary Fig. S2). In all co-infection events where PCV-2d was identified, it persisted longer than other genotypes, suggesting a better fitness of this genotype or, at least, of the particular strain. Because PCV-2d infection occurred after the other genotypes in 2 out of 3 pigs, a still incomplete clearance by the host immune system cannot be excluded. Additionally, a strong recombination signal was detected in the haplotype alignment and RDP4 analysis identified 18 recombinant haplotypes emerging after animal mixing (Table 2).

**Table 2:** Detected recombinant strains and the genotype of the minor and major parental strains are shown together with the potential recombination breakpoints.

Identified Recombinant	Beginning breakpoint	Ending breakpoint	Minor parent	Major parent	Recombination event
745-13_2	386	681	PCV2a	PCV2b	Event 1
748-11_6					
748-11_7					
748-11_5	314	699	PCV2a	PCV2b	Event 2
737-11_1					
738-12_2					
748-14_1					
748-11_0	79	500	PCV2b	PCV2d	Event 3
738-12_4	441	702	PCV2b	PCV2d	Event4
737-11_2					
745-12_1					
748-11_1					
748-11_2					
748-11_8					
738-13_2	81	454	PCV2b	PCV2d	Event 5
737-11_3					
738-12_3					
738-13_3					

## Discussion

PCV-2 is characterized by a high substitution and recombination rate^18^, which has led to the remarkable genetic heterogeneity demonstrated worldwide^9^. Even if overall features of PCV-2 variability have been investigated at epidemiological level, the fine mechanism acting at individual level and their overall impact are still obscure.

The present study, evaluating the within animal viral variability, found a remarkable difference in terms of entropy between the Rep and Cap regions. Such a marked heterogeneity cannot be realistically explained either by a differential viral replication fidelity or by variable NGS error rates. Although the presence of both PCR and sequencing errors together with the complexity in differentiating them from true variations still represents one of the main challenge in the use of NGS for viral subpopulation estimation^19,20^, the consistency of the results obtained from different animals and farms in this study, as well as their biological plausibility, supports the observations described herein. Therefore, a combination of selective constraints acting on proteins involved in viral replication and the presence of multiple ORFs in different orientation and reading frames^1,12^ could limit the plasticity of the Rep coding region by causing most of the mutations to be deleterious and rapidly purged by natural selection. On the other hand, the Cap gene could benefit from a higher genetic plasticity because of the immunological pressures favouring the emergence of immune response evading variants. Capsid entropy demonstrated variable patterns among individuals. Interestingly, several animals showed a fluctuating trend, which was already described for another persisting swine infection like porcine reproductive and respiratory syndrome virus (PRRSV) and ascribed to the interaction with the host immune response^21^. Nevertheless, several exceptions were observed among individual animals and, more generally, an overall different pattern was found between farms A and B. Therefore, the observed scenario is likely the result of a complex network of interactions whose outcome depends on peculiar host and viral strain features as well as environmental conditions not measured here. This high background noise surely hampers the understanding of the underling biological process, and larger studies will be required. Similarly, the individual variability can probably explain the absence of any clear relationship between viral heterogeneity and host immune response. Additionally, the global IgM and IgG titers represent a quite raw measure to investigate the fine interactions between a swarm of viral variants and a population of epitope-specific antibodies and immune system cells. Analogous conclusions can be drawn regarding the evaluation of virus neutralization titers, since different epitopes, antibody classes and lymphocyte clones are likely involved in the phenomena. Nevertheless, despite the limited animal number and the apparent individual peculiarities, several common patterns could be identified.

The existence of *sensu stricto* quasispecies *in vivo* is still highly debated; however, the presence of a complex network of viral subpopulations or quasispecies, evolving within the same individual over time is demonstrated in the present study for the first time for PCV-2. The analysis of the Cap region showed the presence of a major haplotype, typically persisting during the whole study duration, together with other haplotypes that showed variable prevalence and persistence. Comparable results were obtained by the analysis of SNV over time. Therefore, the described scenario is suggestive of the existence of a stable reservoir population from which different evolutionary paths do occur. Even if PCV-2 is recognized as a rapidly evolving virus^18^, the variability herein observed is quite surprising since several mutations were directed towards amino-acidic changes (i.e. leading to phenotypic modifications). In fact, the analysis of selective pressures demonstrated that a neutral to positive selection was primarily acting on the viral capsid. Although the number of animals limited the study power and expose to errors due to random fluctuations, several evidences pose in favor of the results’ robustness. Particularly, several factors were consistently observed on the two farms including similar dN/dS ratios and statistically significant sites. Additionally, all sites detected to be under significant diversifying selection were exposed on the viral surface or, at least, on the capsomer surface, which is compatible with an immune derived immune selection. Therefore, the picture described here enforces the pivotal role of immune derived selective pressure in shaping the within-host PCV-2 diversity. However, the present results are in contrast of other studies demonstrated a purifying selection significantly acting on PCV-2 capsid^12^.

Different biological processes could explain this discrepancy. Firstly, the study duration could have been too short for the removal of variants carrying deleterious mutations, leading to an excess of non-synonymous mutations. Secondly, non-synonymous mutations useful in evading individual pig immune responses could be over specific and negatively affecting the transmission or population level fitness. Interestingly, a similar gap between individual host and population level evolutionary rate has been reported for other viruses^22,23^.

Although a high within-host heterogeneity does not automatically entail a high overall viral fitness, it can represent an effective strategy for within host persistence. At the same time the forces favoring intra-animal diversification can lead to the genesis of some variants that are able to succeed and spread on a broader scale^24^. At least two positions in the capsid (i.e. 68 and 133) proven to be under diversifying selection in the present study, were also reported to be under this kind of selection in other studies investigating PCV-2 evolution^12^ and vaccine escape^25^. Additionally, mutations inside the capsid region at position 133, have been shown to affect cross-reactivity to monoclonal antibodies in experimental studies^26^. Based on these results, the reduction of PCV-2 circulation and persistence, achievable by proper biosecurity measures and vaccination application, appears of major importance since it can constrain the evolutionary potential of this virus and the consequent emergence of variants with different virulence or antigenic/immunological features.

The analysis of viral sub-populations in farm C demonstrated the impact of animal management in herd PCV-2 epidemiology. After animal mixing, the relatively homogeneous PCV-2b population was replaced by a mixed genotype circulation with several co-infection events. The most likely explanation is that animals from different sources were infected with different strains which were rapidly spread among animals. This is clinically relevant for several reasons. At first, co-infections with multiple genotypes have been proposed as a risk factor for clinical signs development^27^ and even when no overt clinical signs were observed as in this study, a negative effect on productive performances is still likely^28^. Co-infection is also a prerequisite for recombination to occur. Several recombinant strains were identified in the present study, all originating after animal mixing. The occurrence of *in silico* recombinants cannot be excluded and represents one of the still unsolved-limits of global haplotype reconstruction in NGS data analysis^29^. However, the virtual simulation made with mixed reads belonging to different genotypes, strongly supports the implemented pipeline ability to accurately reconstruct PCV-2 genotypes minimize *in silico* recombination. The estimated haplotype frequency mirrored the actual ones, set during artificial read generation independently of the selected reference strain genotype, although deviations were observed only when low frequency variants (<5%) were simulated; in this case extremely low frequency haplotypes (<1%) were sometimes missed. Additionally, closely related recombinant sequences were detected in different animals (i.e. independent NGS runs and data analysis), supporting the actual existence of the recombinant forms rather than technical errors, and suggesting a certain fitness of these strains which were able to persist and infect other pigs. Recombination events have been frequently described in different countries and recombinant clusters have been reported to display a fitness and distribution comparable to the “classical” genotypes^12^, representing a potential threat for pig farming. Therefore, demonstrating the high within-host recombination frequency, the present study stresses once more the relevance of adequate control measures to reduce opportunities of recombination and its consequences.

The present study illustrates the remarkable genetic variability of PCV-2 at the individual pig level, particularly in the Cap gene, likely driven by the host immune response. Moreover, this study also points out how farm management (animal flow, mixing of animals) played a major role in the occurrence of multiple genotype co-infections and likelihood of recombination.

## Methods

### Sample collection

Three different farms from the area of Catalonia (Spain) were screened for this study. Sera samples from 20 piglets starting from 3 weeks of age (weaning age), were collected weekly. At 11 weeks of age, all animals from farms A and B were moved to fattening units, remaining in contact only with pigs originating from the same farm in the case of farms A and B. In contrast, animals coming from the third farm (farm C), were mixed with other fattening pigs originating from different herds from the same swine integrator. From 3 weeks of age onwards, serum samples were tested for PCV-2 detection using conventional PCR as previously described^30^. Quantification of PCV-2 DNA in serum was done as described^31^ and IgM and IgG titers were estimated from the sixth week onwards using a commercial Ingezim Circo IgG ELISA kit as recommended (Inmunologia y Genetica Aplicada). A total of 5 infected animals from each farm were selected due to both positive detection of PCV-2 in sera and low maternally derived antibody levels. Longitudinal sampling during four weeks after the first positive PCV-2 detection was performed for each piglet. All methods were carried out in accordance with relevant guidelines and regulations. The present study was approved by the Ethics Committee for Animal Experimentation from the Universitat Autònoma de Barcelona and the Animal Experimentation Commission from the local government (Dpt. de Medi Ambient i Habitatge from the Generalitat de Catalunya; Reference 5796).

### DNA extraction and sequencing

DNA was extracted from these samples using the QiAmp MiniElute Virus Spin Kit (QIAGEN) and the full PCV-2 genome was amplified using the primer PCV2 SacF TCCGCGGGCTGGCTGAACTTTTGA and PCV2 SacR CCCGCGGAAATTTCTGACAAACGT as described elsewhere^32^. Amplicons were purified using the QIAquick PCR purification kit, eluted in 30 µL Tris buffer and both quality and quantity of nucleic acids were evaluated on a BioDrop DUO (BioDrop Ltd). Barcoded libraries were individually constructed for each animal and sampling time at Servei de Genomica Autonomous University of Barcelona using the Nextera^®^ XT DNA Sample Preparation Kit (Illumina^®^, San Diego, CA) and the MiSeq^TM^ Illumina platform (pair-end 2 × 250 bp) following the manufacturer instructions (MS-102-2003 MiSeq® Reagent Kit v2, 500 cycle).

### Data analysis

Read quality of obtained FASTQ files was visually inspected using PRINTSEQ^33^. Reads that fulfilled the following criteria were filtered out, shorter length than 30 nt, average quality lower than 20 or with more than one base with a quality lower than 10. Additionally, adapters and barcodes were removed and trimming of poor quality bases, poly-A/T and poly-N at the 5′ and 3′ tails were performed using the same software.

Overlapping paired reads were merged using mothur^34^ and aligned to the PCV-2 reference genome (NC_005148) using Bowtie2^35^. Samtools^36^ was used to convert, sort and remove duplicates from the obtained SAM files. Remaining reads were extracted and converted to FASTA format using the same software. Finally, sample specific coverage and consensus sequence were obtained using QUASR^37^.

#### Subpopulation study

The analysis of viral subpopulation was conducted at two levels: single nucleotide variation (SNV and entropy calculation) and global haplotype. To limit the bias introduced by the PCR and sequencing error in viral subpopulation estimation, an error correction approach assuming a Poisson distribution of errors parameterized differently in homopolymeric and non-homopolymeric regions was applied in the obtained FASTA sequences^38^ and the corrected read sets were used for further analysis. The number of variations, their coverage and the entropy statistic for each genome position was calculated using the QuRe software^38^. Each position-specific SNV and its frequency was identified with the highly specific LoFreq method^39^, also implementing a Poisson–binomial distribution model and accounting for base-call quality value as well as other sources of uncertainty to identify true variants from sequencing errors. In order to achieve only highly reliable SNV, the significance level was set to p < 0.001. Additionally, global haplotypes and their prevalence were reconstructed using the heuristic algorithm implemented in QuRe, which matches multinomial distributions of distinct viral variants overlapping across the genome division^38^. For each sample, the full genome consensus sequence previously obtained was used as reference. The QuRe software was selected because it was proven to provide a good compromise between sensitivity and false positivity in haplotype reconstruction under experimental conditions^29^. However, to diminish the impact/likelihood of *in silico* recombination and because of the higher phylogenetic signal only the Cap gene was used in the analyses based on global haplotype reconstruction. Additionally, *in silico* simulations were performed to evaluate the accuracy of haplotype reconstruction. Paired reads with features (i.e. read length and error rate) comparable with the experimental setting were simulated using the *wgsim* tool of Samtools^36^. To evaluate the co-infection detection capability, fastq files were generated based on PCV2a, PCV2b and PCV2d genotype reference sequences and merged in different proportions. A similar approach was used to evaluate haplotype reconstruction performances. Particularly, closely related variants were assumed as reference and used to generate simulated reads which were then mixed in variable proportions. All obtained simulated dataset were analyzed with the same pipeline used for the experimental samples and selecting a PCV2a genome as reference.

### Analysis of sub-population evolution and epidemiological relationship

Evidences of recombination between specific haplotypes identified in each farm were assessed using the GARD^40^ method implemented in HyPhy^41^ and the Phi test implemented in Splittree4^42^. When recombination was detected, a further characterization was performed using RDP4^43^, adjusting methods’ settings based on dataset features. Individual haplotypes were genotyped by comparing their ORF2 sequence with the reference dataset proposed by Franzo *et al*.^9^. Sequences were aligned at amino-acid level and then back translated to nucleotide sequences using the MAFFT^44^ method implemented in TranslatorX^45^. Phylogenetic tree was reconstructed using the Maximum likelihood method implemented in PhyML^46^ selecting as substitution model the one with the lowest Bayesian Information Critera, calculated using Jmodeltest^47^. Pig specific haplotype networks were reconstructed using the parsimnet function (which find one of the most parsimonious network within a 95% probability of parsimony as defined in Templeton *et al*.^48^ implemented in the haplotypes R library. The within sample haplotype prevalence was accounted in the analysis. The presence of selective forces acting on the Cap gene of the identified variants was evaluated on farm-specific alignments using the MEME^49^, FUBAR^50^, SLAC and FEL^51^ methods implemented in HyPhy^41^. Less stringent significance value (p-value < 0.1) of posterior probability (>0.9) were accepted considering the limited sequence number.

### Relationship between genetic variability and Ab

Potential interaction between host antibody response and viral evolution was globally evaluated by comparing the average entropy with antibody levels at different wpi. Considering the specificity of host immune response toward specific regions of viral proteins, a more focused analysis was performed using a sliding window approach: the windows and step sizes were set to 40 and 20 nucleotides, respectively. The relationship between antibody titer and genetic variability was assess by calculating the correlation between mean entropy in the specific windows region and the antibody titer (IgM and IgG). Also, all sera samples were tested for their ability to neutralize PCV-2 by an *in vitro* neutralization assay. The virus neutralization assay was performed as described previously^52^. Data obtained from all animals and wpi were used in the correlation calculation.

## Electronic supplementary material

Supplementary figures

Supplementary Video

## Data Availability

The entire sequence dataset is available in the NCBI database, BioProject PRJNA450261 (available at http://www.ncbi.nlm.nih.gov/bioproject/450261).
