# Supplementary material for: Porcine circovirus 2 (PCV-2) genetic variability under natural infection scenario reveals a complex network of viral quasispecies
Source: Sci Rep. 2018 Oct 19;8:15469. doi: 10.1038/s41598-018-33849-2 (PMC6195574; doi:10.1038/s41598-018-33849-2)
Supplement: Supplementary file 1 — Supplementary figures [file 41598_2018_33849_MOESM1_ESM.pdf]

Porcine circovirus 2 (PCV-2) genetic variability under natural infection scenario reveals a complex network of viral quasispecies.

Florencia Correa-Fiz[a*], Giovanni Franzo[b], Anna Llorens[a], Joaquim Segalés[a,c], Tuija Kekarainen[a#]

[a]Centre de Recerca en Sanitat Animal (CReSA, IRTA-UAB), IRTA, Bellaterra, Spain

[b]Department of Animal Medicine, Production and Health (MAPS), University of Padua, Legnaro (PD), Italy.

[c]Departament de Sanitat i Anatomia Animals, Facultat de Veterinària, UAB, Bellaterra, Spain

Running Head: PCV-2 variability under natural infection scenario.

*Address correspondence to Florencia Correa-Fiz, flor.correa@irta.cat.

#Present address: Tuija Kekarainen, Kuopio Center for Gene and Cell Therapy, Microkatu 1, Kuopio, Finland.

F.C.F. and G.F. contributed equally to this work.

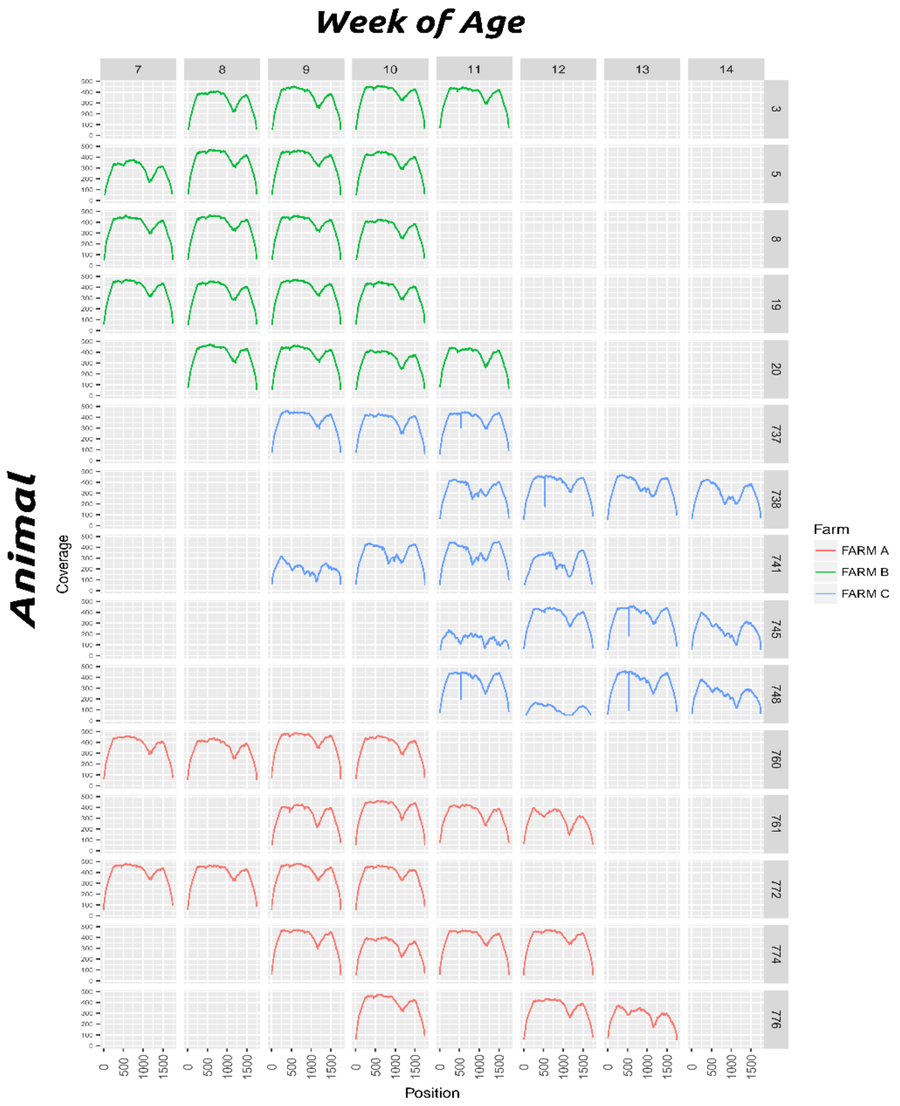

Supplementary Fig. S1. Sequencing depth at each sampling point associated to each genome position. Columns and rows code for animal age (in weeks) and ID, respectively. Different farms have been color-coded.

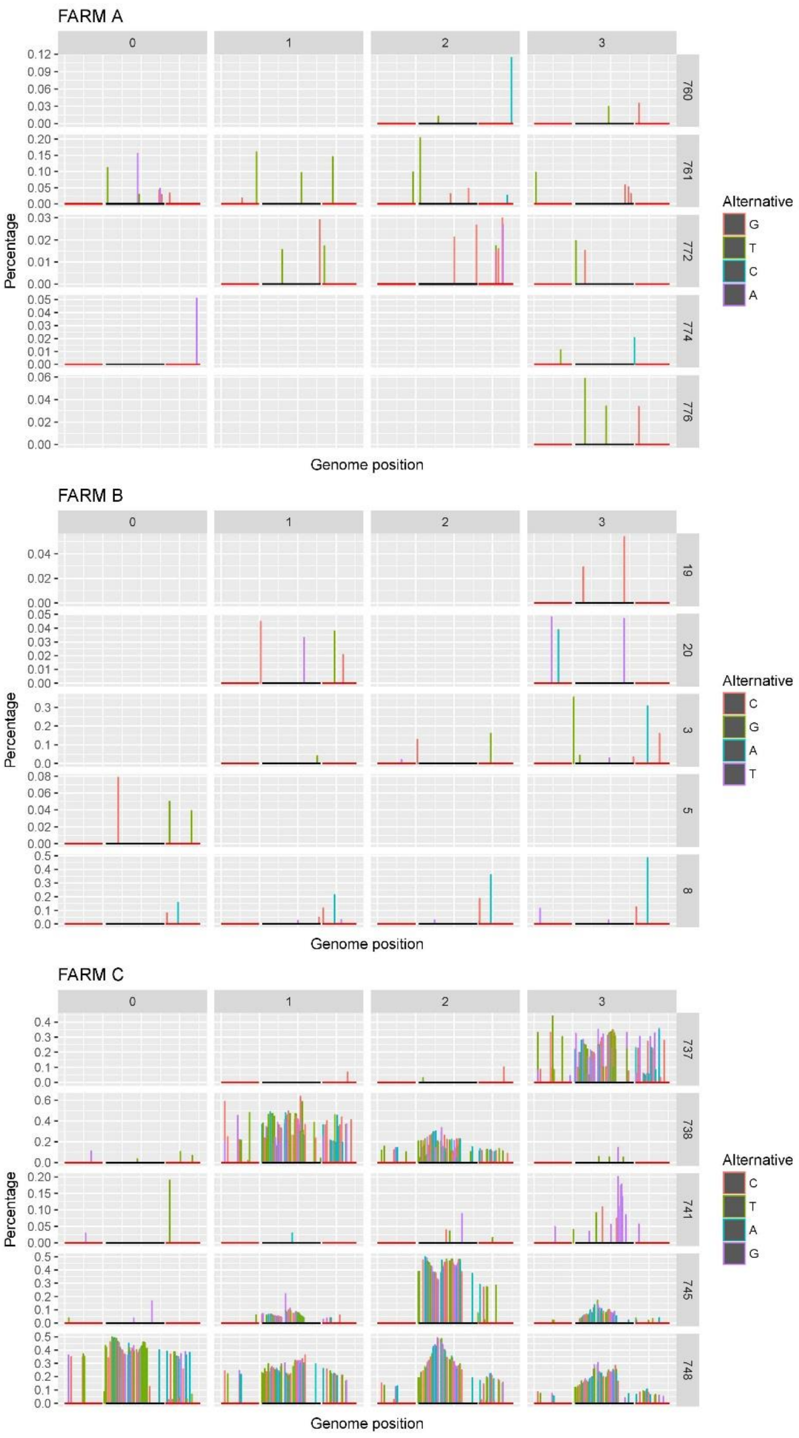

Supplementary Fig. S2. Minority variants nucleotide frequency (i.e. frequency lower than 50%) at each genome position are reported for farm A (top), B (middle) and C (bottom). Data for different animal and week post infection (wpi) have been reported separately. Different nucleotides have been color coded. The ORF1 and ORF2 have been respectively represented as red and black line in the lower part of each graph.

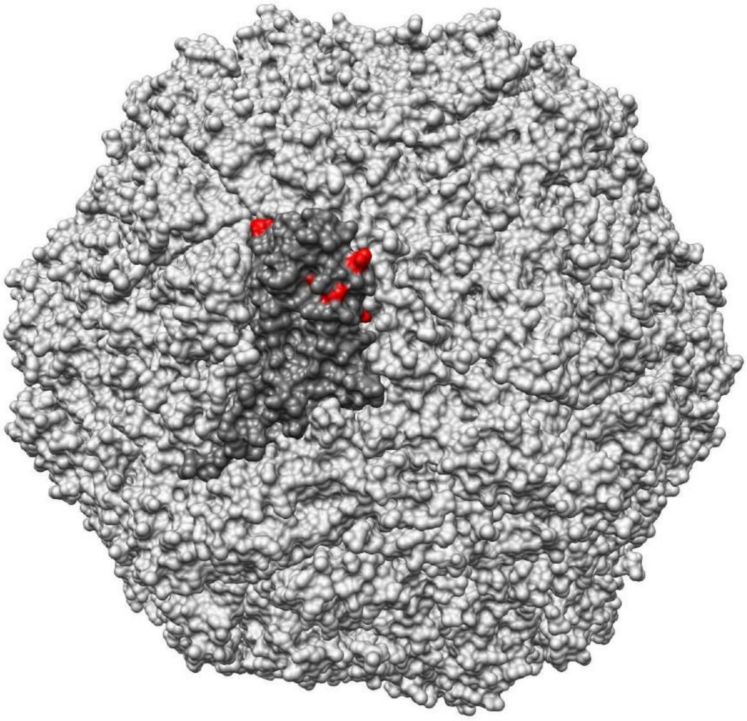

Supplementary Fig. S3. Quaternary structure reconstruction of the PCV-2 viral capsid. Sites detected to be under significant diversifying selection have been highlighted in red.

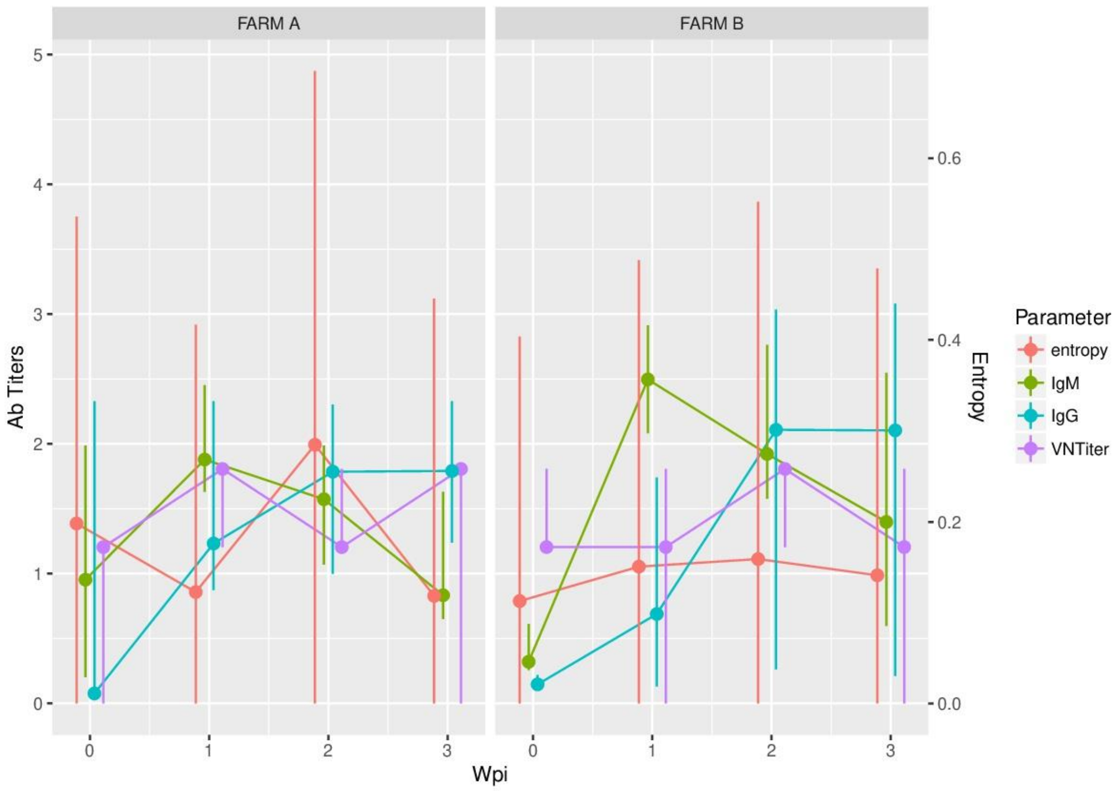

Supplementary Fig. S4. Median (point) and percentile range (error-bars) excluding the outer 25% values of IgM, IgG (expressed in OD 450nm), virus neutralization tiers (VN, expressed in log10 of antibody titre) and entropy (Sh) are reported for each week post infection (wpi). Although not comparable in absolute value, all the results have been provide to graphically show the relative trends.

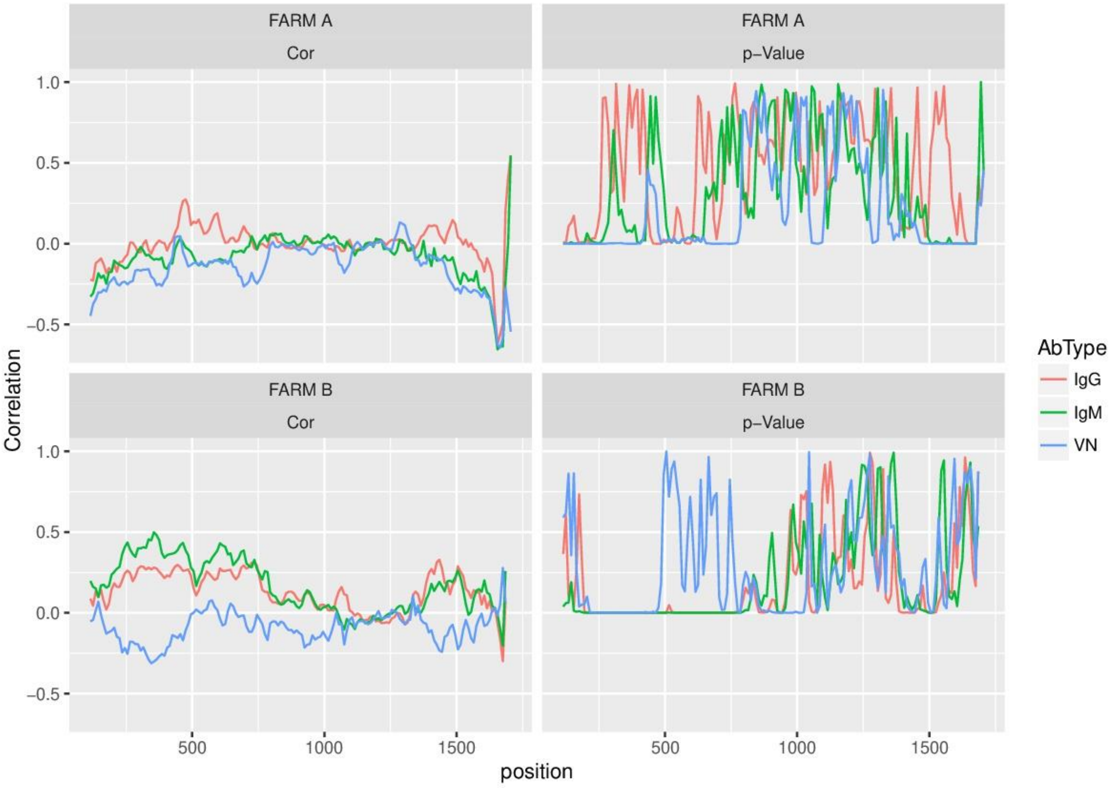

Supplementary Fig. S5. Correlation calculated between entropy and IgM, IgG or virus neutralization (VN) titers for each considered genome window. The statistical significance of the detected correlation is also reported.
